# Milk and dairy products consumption and the risk of oral or oropharyngeal cancer: a meta-analysis

**DOI:** 10.1042/BSR20193526

**Published:** 2019-12-16

**Authors:** Jian Yuan, Wen Li, Wei Sun, Shuli Deng

**Affiliations:** 1 Department of Conservative Dentistry and Endodontics, Stomatological Hospital Affiliated to Medical College, Zhejiang University, Hangzhou 310006, Zhejiang Province, China; 2 Department of Orthodontics, Stomatological Hospital Affiliated to Medical College, Zhejiang University, Hangzhou 310006, Zhejiang Province, China; 3 Stomatological Hospital Affiliated to Medical College, Zhejiang University, Hangzhou 310006, Zhejiang Province, China

**Keywords:** dairy products, meta-analysis, milk, oral cancer, oropharyngeal cancer

## Abstract

The present meta-analysis was conducted to explore the role of milk and dairy products consumption on oral or oropharyngeal cancer risk. PubMed, Embase and Chinese Wanfang databases were investigated until 30 June 2019. The overall and subgroup associations were pooled with odds ratios (ORs) and 95% confidence intervals (CIs). As a result, the present study involving 4635 cases and 50777 participants from 12 publications suggested that an inverse association was found between milk and dairy products consumption and oral or oropharyngeal cancer risk (OR = 0.74, 95% CI = 0.59–0.92; *I^2^* = 65.9%, *P*_for heterogeneity_=0.001). Four studies reported milk consumption on oral cancer risk, but no significant association was found (OR = 0.91, 95% CI = 0.61–1.37). Six studies about milk consumption and oropharyngeal cancer risk found that there was a positive association between them (OR = 0.63, 95% CI = 0.44–0.90). In conclusion, findings from our meta-analysis indicated that milk and dairy products consumption may be associated with decreased risk of oral or oropharyngeal cancer.

## Introduction

Oral cavity and pharyngeal cancer, which includes cancer from the tongue, buccal mucosa, upper and lower gums, mouth and hard palate, pharynx, is the eighth most common cancer in the world [1]. A previous paper showed that oral cancer may account for approximately 34000 new cases and 7000 deaths in United States in 2018 [1]. Therefore, primary prevention is important to reduce the incidence and mortality of oral cavity and pharyngeal cancer. Many researchers have explored the association of dietary factors and the risk of oral or oropharyngeal cancer, such as tea consumption [2], fruit and vegetable consumption [3], coffee consumption [4], meat consumption [5]. But, there is no meta-analysis about milk and dairy products consumption and oral or oropharyngeal cancer risk. Notani and Jayant [6] first explored the association about milk consumption of the risk of oral cancer in 1987. Since then, more studies had attempted to assess their relevance. However, no exact conclusion was obtained probably due to the small sample size and inadequate statistical power in each individual study. Thus, the current study used a meta-analysis to pool as many eligible studies as possible in the relevant database to get a more exact result about milk and dairy products consumption and oral or oropharyngeal cancer risk.

## Methods

### Identification of eligible studies

As many eligible studies as possible in the relevant database about milk consumption and oral cancer risk were investigated by two independent authors (J.Y. and W.L.) until 30 June 2019. The databases included PubMed, Embase and Chinese Wanfang database. The associated keywords were (‘milk’ or ‘dairy’) combined with (‘oral cancer’ or ‘oral cavity cancer’ or ‘oral oncology’ or ‘oropharyngeal cancer’ or ‘pharyngeal cancer’). Meanwhile, we also manually searched for the references which cited in the eligible articles or reviews to identify relevant studies. Disagreement was resolved by a third reviewer (W.S.).

The inclusion criteria were as follows: (1) observational study; (2) assessed the association of milk and dairy products consumption and oral or oropharyngeal cancer risk; (3) provided odds ratios (ORs) and 95% confidence intervals (95% CIs) or enough information for calculating them in independent study; (4) reported in humans.

The exclusion criteria were as follows: (1) case reports, conference abstracts, letters, editorials, reviews; (2) overlapping or duplicate studies; (3) irrelevant studies; (4) no available data of OR and 95% CI.

### Data extraction and quality assessment

Information which was provided in Table 1 from all included publications was independently extracted by two investigators (J.Y. and W.L.). Disagreement was resolved by a third reviewer (W.S.). The Newcastle–Ottawa Scale (NOS) was used for evaluating the quality of each study [7].

**Table 1:** Characteristics of the included studies

Study, year	Design	Age (years)	Country	Participants, Cases	Dietary intake	Outcomes	Quality score	Category	OR (95% CI)	Adjusted for or matched for
Bravi et al., 2013	HBCC	22–79	Switzerland	2846, 768	Milk and yoghurt	Oropharyngeal cancer	7	Q4 vs. Q1	0.98 (0.70–1.38)	Age, sex, centre, education, year of interview, body mass index, tobacco smoking, alcohol drinking and nonalcohol energy intake
Chen et al., 2017	HBCC	20–80	China	3597, 930	Milk and dairy products	Oral cancer	7	≥1 time/week vs. <1 time/week	0.63 (0.53–0.74)	Age, gender, education, residence, BMI, family history of cancer, tobacco smoking, alcohol consumption, denture wearing, recurrent oral ulceration
De Stefani et al., 1994	HBCC	40–89	Uruguay	499, 246	Milk	Oropharyngeal cancer	6	≥14 times/week vs. <7 times/week	1.1 (0.6–1.8)	Age, residence, education, pack-years and total alcohol consumption
Fernandez Garrote et al., 2001	HBCC	28–91	Cuba	400, 200	Milk	Oropharyngeal cancer	7	≥7 times/week vs. <1 time/week	0.72 (0.39–1.33)	Age, sex, area of residence, education, and smoking and drinking habits
Gallus et al., 2006	HBCC	22–77	Italy	2089, 598	Milk	Oropharyngeal cancer	7	>7 times/week vs. <7 times/week	0.84 (0.61–1.33)	Age, sex, centre, education, smoking habit, alcohol and energy intake
La Vecchia et al., 1991	HBCC	37–74	Italy	1274, 105	Milk	Oropharyngeal cancer	6	>7 times/week vs. <1 time/week	0.3 (0.2–0.7)	Age, sex
Levi et al., 1998	HBCC	26–72	Switzerland	440, 156	Milk	Oropharyngeal cancer	7	T3 vs. T1	0.38 (0.21–0.70)	Age, sex, education smoking, alcohol and total energy (other than alcohol) intake
Lissowska et al. 2003	HBCC	23-80	Poland	246, 122	Milk	Oral cancer	8	≥7 times/week vs. <2 times/week	0.41 (0.19–0.89)	Age, sex, race, residence, tobacco use (smoking habits), alcohol consumption
Notani et al., 1987	HBCC	30–70	India	670, 278	Milk	Oral cancer	7	≥7 times/week vs. <7 times/week	0.87 (0.47–1.67)	Age, sex and tobacco habits
Rajkumar et al., 2003	HBCC	18–87	India	1173, 591	Milk	Oral cancer	7	≥5 times/week vs. never	1.14(0.70-1.87)	Age, sex, centre, education, chewing, smoking and drinking habits.
Sanchez et al., 2003	HBCC	20–91	Spain	750, 375	Milk	Oropharyngeal cancer	8	≥9 times/week vs. ≤6 times/week	0.67 (0.44–1.01)	Age, gender, centre, years of schooling, smoking and drinking habits
Takezaki et al. 1996	HBCC	20–79	Japan	36793, 266	Milk	Oral cancer	7	T3 vs. T1	1.2 (0.8–1.8)	Age, sex, smoking, drinking and year of visit

Abbreviations: BMI, body mass index; HBCC, hospital-based case–control study; Q1, quartile 1; Q4, quartile 4; T1, Tertile 1; T3, Tertile 3.

### Statistical analysis

Pooled association of milk and dairy products consumption and oral or oropharyngeal cancer risk was calculated with OR and 95% CI [8]. The *I^2^* was used to evaluate the heterogeneity and *I^2^* > 50% was considered as significant heterogeneity [9,10]. A random-effect model was used on the overall and subgroup analyses. Sensitivity analysis was performed to explore whether a single study had significant impact on the pooled OR. Publications bias was detected using Begg’s funnel plots [11] and Egger’s test [12]. All analyses in the current study were calculated with Stata 10.0 software (College Station, TX). *P*<0.05 was set as statistically significant.

## Results

### Researches characteristics

**Figure 1:**
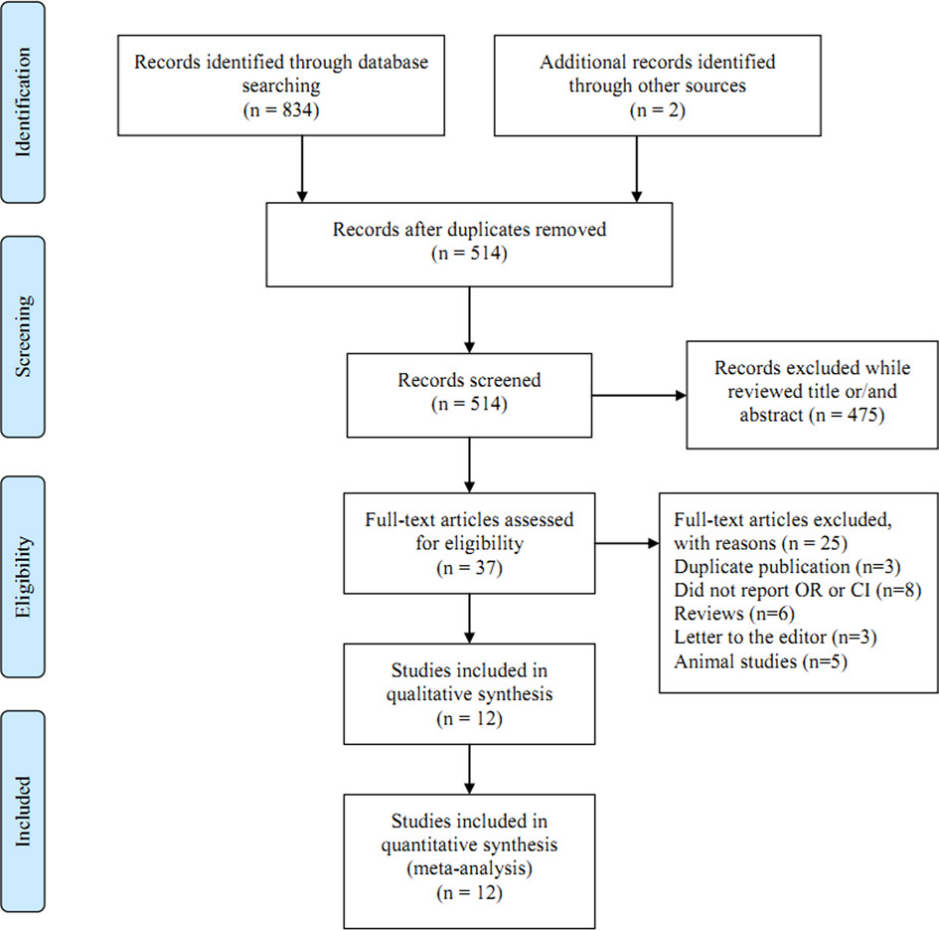
Flow chart of meta-analysis for exclusion/inclusion of studies

Figure 1 shows the process of included or excluded reason. Totally, 834 relevant publications (396 papers from PubMed, 372 papers from Embase and 66 papers from Chinese Wanfang database) about milk and dairy products consumption and oral or oropharyngeal cancer risk were searched. At last, 12 papers [6,13–23] including 4635 cases and 50777 participants were used in the final analysis. Six studies were from Europe, four from Asia and two from America. All included studies were with case–control design. All the nine studies had relatively high quality (over 6 stars), with an average NOS score of 7. Table 1 presents the characteristics of each individual study.

### Meta-analysis results

**Figure 2:**
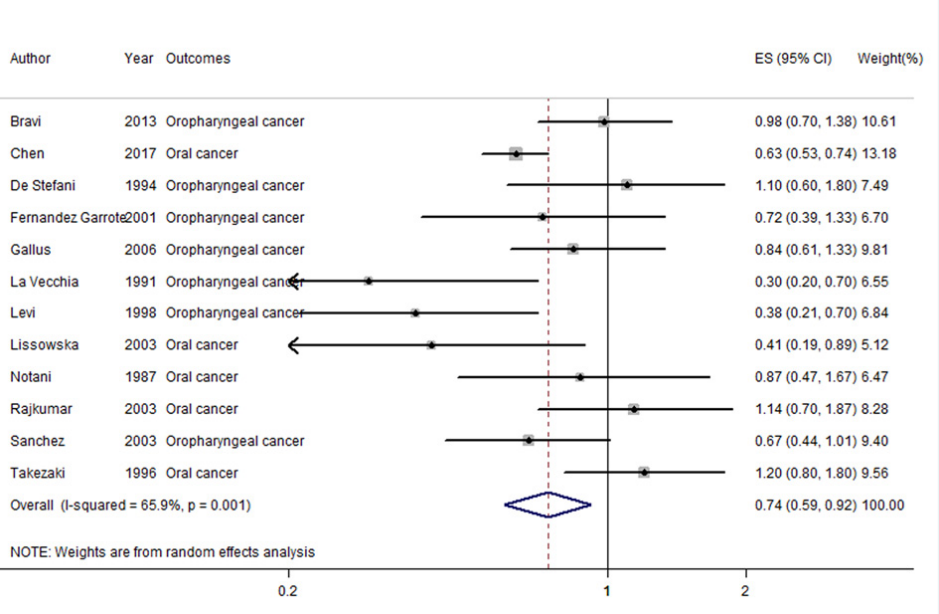
The forest plot of the association about milk and dairy products consumption on the risk of oral or oropharyngeal cancer

Pooled result suggested that an inverse association was found between milk and dairy products consumption and oral or oropharyngeal cancer risk (OR = 0.74, 95% CI = 0.59–0.92; *I^2^* = 65.9%, *P*_for heterogeneity_=0.001) (Figure 2).

**Figure 3:**
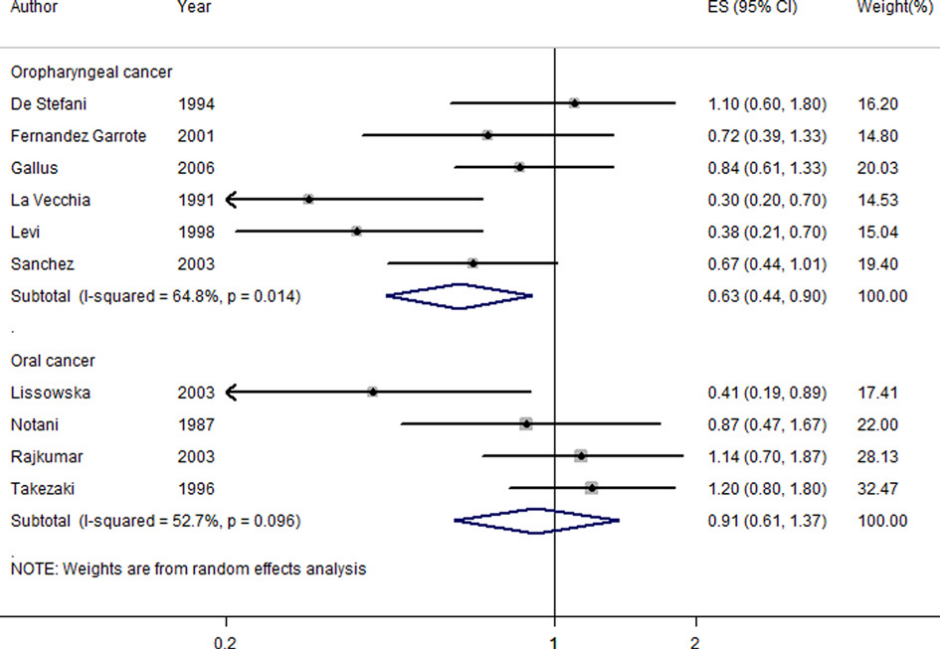
The forest plot of the association of milk consumption on the risk of oral cancer and oropharyngeal cancer independently

Four studies [6,20,21,23] reported milk consumption on oral cancer risk, but no significant association was found (OR = 0.91, 95% CI = 0.61–1.37) (Figure 3). Six studies [15–19,22] about milk consumption and oropharyngeal cancer risk found that there was a positive association between them (OR = 0.63, 95% CI = 0.44–0.90) (Figure 3). Chen et al. [14] reported the association about milk and dairy products on oral cancer risk. Bravi et al. [13] assessed the association of milk and dairy products on oropharyngeal cancer risk. Therefore, we did not combine the last two studies [13,14].

When we explored the association of milk and dairy products consumption and oral or oropharyngeal cancer geographic location, significant association was only found in European populations (OR = 0.58, 95% CI = 0.40–0.85; *I^2^* = 71.2%, *P*_for heterogeneity_=0.004), but not in Asian populations (OR = 0.90, 95% CI = 0.61–1.34; *I^2^* = 75.5%, *P*_for heterogeneity_=0.007) or in American populations (OR = 0.91, 95% CI = 0.60–1.38; *I^2^* = 1.7%, *P*_for heterogeneity_=0.313).

We also considered the effect of age and smoking, which may affect the risk of oral or oropharyngeal cancer except ethnicity. All the included studies were adjusted for age, and the result was consistent with the overall result. Ten of the twelve included studies were adjusted with smoking. The ORs in adjusted for smoking and not adjusted for smoking were 0.77 (95% CI = 0.62–0.95) and 0.58 (95% CI = 0.16–2.07), respectively.

### Sensitivity analysis and publication bias

**Figure 4:**
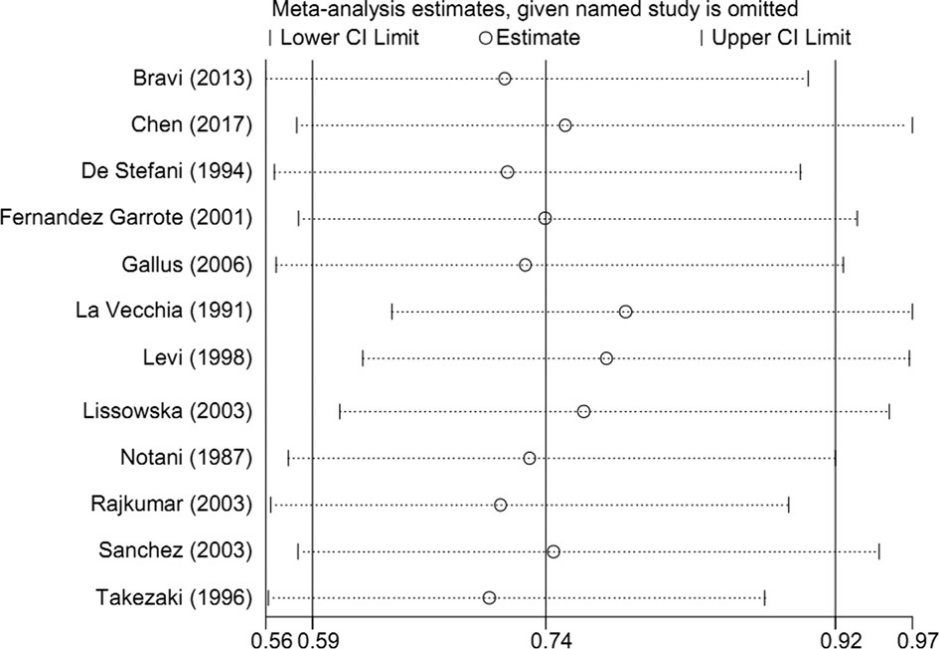
Sensitivity analyses between milk and dairy products consumption and the risk of oral or oropharyngeal cancer

**Figure 5:**
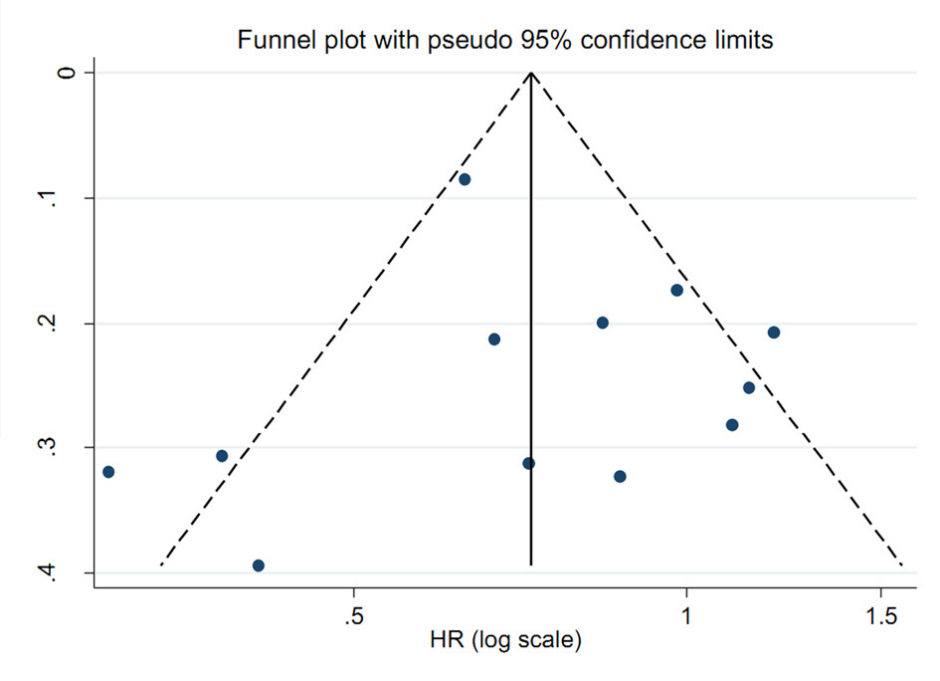
Funnel plots about milk and dairy products consumption on the risk of oral or oropharyngeal cancer

Sensitivity analysis (Figure 4) showed that no single study had essential impact on the overall result while removed a study sequentially. There was no significant publication bias detected by funnel plots (Figure 5) and Egger’s test (*P*=0.868).

## Discussion

Whether milk and dairy products consumption is associated with reduced risk of oral or oropharyngeal cancer is still attracting attention from researchers. So far, no meta-analysis had been performed regarding milk and dairy products consumption on oral or oropharyngeal cancer risk. Thus, the current meta-analysis was carried out to pool as many eligible studies as possible that met our inclusion criteria in the relevant database. Our meta-analysis included 12 studies, comprising 4635 cases and 50777 participants. Findings from our study indicated that milk and dairy products consumption may reduce the risk of oral or oropharyngeal cancer.

To our attention, significant heterogeneity was found in the overall analysis. Though between-study heterogeneity is common in a meta-analysis, we did our best to explore the sources of heterogeneity. Meta-regression was first performed to evaluate if this high heterogeneity was caused by publication years, geographic locations, study design or sample size. Results from meta-regression suggested that all the above-mentioned factors were not associated with this high heterogeneity. We then performed subgroup analyses by geographic location and study design to further explore the sources of heterogeneity. However, the heterogeneity was significant in subgroup analyses. Thus, some other factors, such as genetic or environmental or their interaction may affect the development of oral cancer.

Ethnicity, age, smoking etc, may affect the development of oral cancer. Results from our study suggested that milk and dairy products consumption could decrease the risk of oral or oropharyngeal cancer only in European populations, instead of other populations, this may be due to the habit of drinking milk or different levels of milk consumption. All the included studies were adjusted for age, and the result was consistent with the overall result. Smoking may be another risk factor for oral cancer [24]. In our results, only the study which adjusted for smoking obtained an inverse association between milk and dairy products consumption and the risk of oral or oropharyngeal cancer. Therefore, the study was suggested to adjust for smoking until further analysis.

Milk and dairy products consumption had been studied on risk of many cancers [25–27]. The mechanism of milk and dairy products consumption on the risk of oral or oropharyngeal cancer is still unclear. Milk contains high-quality protein, which can enhance the body’s immunity and promote the body to return to health. Therefore, drinking milk and dairy products may protect against the oral or oropharyngeal cancer. In our study, we also obtained an inverse association about milk and dairy products consumption and oral or oropharyngeal cancer risk.

Some limitations existed in the current study should be noticed. First, we only pooled the overall results about milk and dairy products consumption and oral or oropharyngeal cancer risk. To our knowledge, milk contains many different types, such as whole milk, low-fat milk, nonfat milk. Different types of milk may affect the role on the risk of oral or oropharyngeal cancer. However, there is no detailed information about milk types in each individual study. Thus, more studies with different milk types are warranted to further confirm these results. Secondly, all the included studies are case–control design. As far as we knowledge, case–control studies may cause some bias, such as selected bias, recalling bias and so on. Therefore, more cohort studies are needed to further explore the association of milk and dairy products consumption and risk of oral or oropharyngeal cancer. Third, when we performed the subgroup analysis of milk consumption on oral cancer or oropharyngeal cancer independently, we only found an inverse association on oropharyngeal cancer, but not on oral cancer. This may be affecting by the small number of included studies of milk consumption on oral cancer risk. Fourth, the level of milk consumption in each included study is very different, which may contribute to the between-study heterogeneity and affect the overall result. However, we are currently unable to control the level of milk consumption because the OR value about milk and dairy products consumption on oral or oropharyngeal cancer risk in each study corresponds to a different category of milk consumption, such as ≥1 time/week vs. <1 times/week, ≥14 times/week vs. <7 times/week. Therefore, more detailed level of milk consumption in the individual study should be clarified, and then we can perform a dose–response analysis with milk consumption on the risk of oral cancer.

## Conclusion

In summary, our meta-analysis indicated that milk and dairy products consumption may be associated with decreased risk of oral or oropharyngeal cancer. While some limitations existed in our meta-analysis, further studies with large participants are warranted to confirm this association.

## Abbreviations

CI: confidence interval
NOS: Newcastle–Ottawa Scale
OR: odds ratio
